# Atomistic Analysis of ToxN and ToxI Complex Unbinding Mechanism

**DOI:** 10.3390/ijms19113524

**Published:** 2018-11-09

**Authors:** Guodong Hu, Xiu Yu, Yunqiang Bian, Zanxia Cao, Shicai Xu, Liling Zhao, Baohua Ji, Wei Wang, Jihua Wang

**Affiliations:** 1 Shandong Key Laboratory of Biophysics and Institutes of Biophysics, Dezhou University, Dezhou 253023, China; m13723922800@163.com (X.Y.); bianyunqiang@163.com (Y.B.); qiayilai@mail.ustc.edu.cn (Z.C.); xushicai001@163.com (S.X.); zhaoll@sina.com (L.Z.); jbh1971@126.com (B.J.); 2 National Laboratory of Solid State Microstructure and Department of Physics, Nanjing University, Nanjing 210093, China; wangwei@nju.edu.cn

**Keywords:** ToxIN, unbinding mechanism, molecular dynamics simulation, steered molecular dynamics simulation

## Abstract

ToxIN is a triangular structure formed by three protein toxins (ToxNs) and three specific noncoding RNA antitoxins (ToxIs). To respond to stimuli, ToxI is preferentially degraded, releasing the ToxN. Thus, the dynamic character is essential in the normal function interactions between ToxN and ToxI. Here, equilibrated molecular dynamics (MD) simulations were performed to study the stability of ToxN and ToxI. The results indicate that ToxI adjusts the conformation of 3′ and 5′ termini to bind to ToxN. Steered molecular dynamics (SMD) simulations combined with the recently developed thermodynamic integration in 3nD (TI3nD) method were carried out to investigate ToxN unbinding from the ToxIN complex. The potentials of mean force (PMFs) and atomistic pictures suggest the unbinding mechanism as follows: (1) dissociation of the 5′ terminus from ToxN, (2) missing the interactions involved in the 3′ terminus of ToxI without three nucleotides (G31, A32, and A33), (3) starting to unfold for ToxI, (4) leaving the binding package of ToxN for three nucleotides of ToxI, (5) unfolding of ToxI. This work provides information on the structure-function relationship at the atomistic level, which is helpful for designing new potent antibacterial drugs in the future.

## 1. Introduction

Bacteria have evolved various phage-resistance mechanisms to resist bacteriophage infection [1]. Resistance strategies include the abortive infection (Abi) systems [2], which promote cell death and limit phage replication within a bacterial population. Abi can be mediated by a toxin–antitoxin (TA) pair [3,4], which is widespread throughout prokaryotes [5]. The TA pairs in bacteria maintain a stable dynamic balance in a common environment. When bacteria are in an adverse environment, the antitoxin with less stability is preferentially degraded in response to stimuli, which leads to toxin release from compound. Then, the free toxins in the cells can lead to bacterial altruistic death or resistance. TA pairs are classified into five types based on the mechanism by which the antitoxin inhibits toxin activity [6,7,8]. All share common features, in that they include a toxic protein that is usually bicistronic in architecture, but are distinguished from each other by the manner in which this toxic protein is antagonized [9]. In Type III, a small RNA interacts directly with the protein toxin to block its activity [3,10].

**Figure 1:**
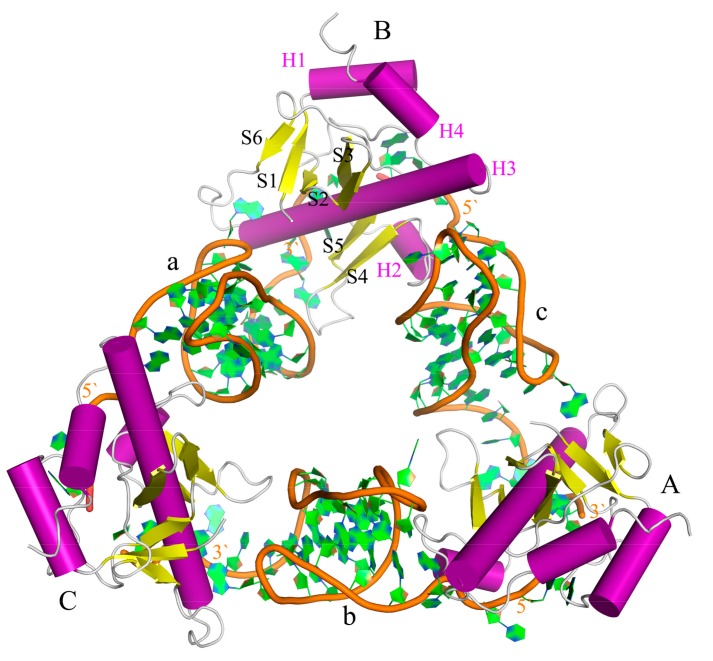
Structure of three protein toxins and three noncoding RNA antitoxins complex (ToxIN) shown in cartoon representation. Three ToxNs (protein toxins) and ToxIs (specific RNA antitoxins) are labeled with uppercase and lowercase letters, respectively. Secondary structures of ToxN(B) are also labeled with α-helices H1 to H4 and β-strands S1 to S6.

The first example of type III is the ToxIN system which is encoded by a cryptic plasmid of *Erwinia carotovora* subsp. *atroseptica*, pECA1039. This system consists of a protein toxin (ToxN) and a specific RNA antitoxin (ToxI) [3,4]. The crystal structure of the ToxIN complex determined by Blower et al. from *Pectobacterium atrosepticum* [11] is a triangular structure formed by extensive cross-interaction with a ToxN at each vertex of the triangle (Figure 1). ToxN has 162 amino acids, consisting of four alpha helices (H1–H4) and six antiparallel beta folds (S1–S6). ToxI, a 36-nucleotide noncoding RNA, folds as an interdigitated hairpin-type pseudoknot, seems to be vital for inhibition of the toxin and is entirely unique throughout known examples of TA systems [11].

RNA pseudoknots, one of the most prevalent motifs, have been found to play a variety of diverse roles in biology. These roles include forming the catalytic core, self-splicing introns, and altering gene expression by inducing ribosomal frame shifting in many viruses [12,13,14,15]. Moreover, pseudoknots have been artificially developed to bind specific small molecules [16] and even to inhibit proteins such as the HIV-1 reverse transcriptase [17]. G5 is a key determinant of the pseudoknot fold. Mutagenesis studies have corroborated its importance by the loss of antitoxicity with a ToxI G5A mutation [11].

The crystallographic structure shows extensive interactions between ToxN and ToxI, which play a vital role for abortive infection activity and maintenance of the complex in the cell. As suggested by Blowser et al. [11], evaluating the binding model ToxN and ToxI would be helpful to understand the protein-noncoding RNA pseudoknot interactions and potentially exploit the specific molecular recognition for therapeutic applications. Understanding how the ToxIN complex accomplishes its functions will necessarily involve the understanding of interactions and binding/unbinding at the atomistic level [18]. Molecular dynamics (MD) simulations could serve as a powerful tool for understanding the structure-function relationship of macromolecules [19,20,21]. Estarellas et al. have carried out a series of explicit solvent MD simulations of the Csy4/RNA RNP complex [22]. Wang’s group have investigated the binding-induced conformational changes in the recognition of the 5S RNA by the three central zinc fingers of protein TFIIIA [23]. Molecular dynamics simulations test minute changes, ascertain the dynamical properties of the system, and provide more accurate structural ensembles [24,25,26,27,28,29]. Even so, it is still difficult for equilibrated MD simulation to observe large conformational changes, especially for the unbinding of large complexes. Steered molecular dynamics (SMD) simulations take inspiration from single-molecule pulling experiments [30], and dissociate a complex structure by a pulling force [31,32]. SMD simulations have become widely used in studying many biochemical processes, including the transportation of ions and organic molecules across membrane channels [33,34,35,36,37] and protein unfolding/folding mechanisms [38], as well as the mechanisms of protein–ligand binding [31,39,40,41].

In the current work, we carried out three 1 µs MD simulations on ToxI, ToxN, and their complex. The conformational differences of ToxI and ToxN in monomer and in complex were investigated by the root mean square deviations (RMSDs) and principal component (PC) analyses. We also studied the function of nucleotide G5 in ToxI by the comparison of wild-type ToxI and its two mutations of G5. Steered molecular dynamics (SMD) simulations and potentials of mean force (PMFs) were used to evaluate the unbinding of ToxIN.

## 2. Results and Discussion

### 2.1. Stability of the Systems

**Figure 2:**
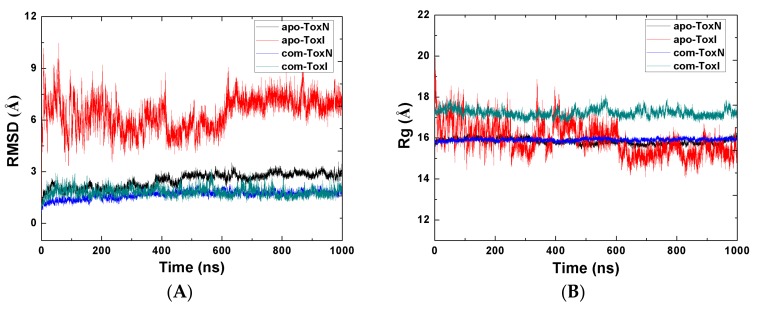
(**A**) The root mean square deviations (RMSDs) of the selected atoms with respect to their crystal structures as a function of molecular dynamics (MD) simulation time. (**B**) Radius of gyration as a function of MD simulation time. The prefix “apo” refers to the monomer form, and “com” refers to the complexed form.

To evaluate the stability of the MD simulations, we calculated the RMSD values of these structures in MD simulations with respect to their original structures. Figure 2 shows the RMSDs and the radius of gyration (Rg) versus the MD simulation time for one of three MD simulations. This confirms that all systems were in stable states after 700 ns MD simulation time. The last 300 ns of MD trajectories were used for subsequent analysis. The comparative plots of the RMSDs reveal that the RMSD values for ToxN were significantly larger in monomer (apo-ToxN) than in complex (com-ToxN), and the same was true for ToxI. The fluctuation of the RMSD values were also much higher in the apo-ToxI and apo-ToxN (*σ* = 0.47 and 0.16) than in the com-ToxI and com-ToxN (*σ* = 0.24 and 0.12). On the other hand, the average values of Rg of ToxI for monomer (*σ* = 0.17) were smaller than those for complex (*σ* = 0.41), suggesting a compact and stable change from complex to monomer. Then, apo-ToxI underwent large conformational changes during the MD simulation process starting from com-ToxI conformation, which is also in agreement with the fact that the protein or RNA do not necessarily adopt their lowest potential energy conformation when forming a complex. ToxI is a pseudoknot with two single-stranded tails interacting with ToxN in complex. To find out which parts of ToxI change, we calculated the RMSDs of ToxI for nucleotides from 5 to 31. The average RMSD over the last 300 ns MD simulation trajectories for the whole ToxI (7.04 Å) were much larger than for ToxI (5–31) (2.34 Å), indicating that two tails changed substantially relative to the pseudoknot in the monomer.

PC analyses were also performed based on the last 300 ns MD simulation trajectory. Figure S1 shows plots of the eigenvalues obtained from the diagonalization of the covariance matrix of the fluctuations of chosen atoms Cα for ToxN and atoms (P, O3′, O5′, C3′, C4′, C5′) for ToxI, depicted in decreasing order versus the corresponding eigenvector indices. The magnitudes of the eigenvalues of ToxI and ToxN in bound state were lower. The first five principal components (PCs) accounted for 54.7%, 49.7%, 78.2%, and 68.0% for apo-ToxN, com-ToxN, apo-ToxI, and com-ToxI, respectively. The first eigenvalues, which are relative to concerted motions, decreased quickly in amplitude and reached a constrained number, indicating more localized fluctuations rather than global fluctuations in the MD simulations.

### 2.2. The Interaction between ToxI and ToxN

**Figure 3:**
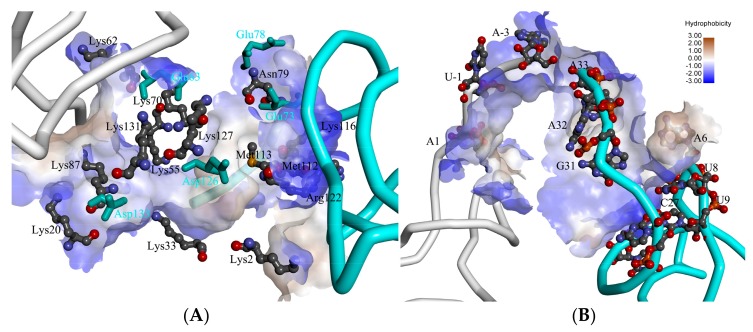
The key residues (**A**) and nucleotides (**B**) for the ToxN and ToxI interaction. The interaction surfaces are shown in hydrophobic surface. The structures of ToxN and ToxI are shown in white or pale cyan colors with tube representation. The key residues and nucleotides with favorable contribution are shown in ball and stick representation, and residues with unfavorable contribution are shown in stick representation with cyan color.

To evaluate the interaction between ToxI and ToxN from the energetic viewpoint, we have calculated the binding free energy between each residue and ToxI, as well as between each nucleotide and ToxN by utilizing the free energy decomposition method. The key residues and nucleotides are shown in Figure 3. Fourteen residues of ToxN interacted with ToxI with large favorable energies and five residues with unfavorable energies |∆*G_residue-ToxI_*| ≥ 5 kcal/mol (Table S1). These residues were located at the interaction surface between ToxN and ToxI (Figure 3A). Note that these residues were either polar or charged, with the exception of Met11. The decomposition method demonstrates that the main energy terms of these residues are from the electrostatic 3 contribution, except Met113 is mainly driven by the van der Waals energy. The charged residues are divided into two groups. One group has positive charges including lysine and arginine residues, which provide a favorable contribution. The other group including negative charge aspartic acid and glutamic acid contribute unfavorably. These polar or charged residues form a hydrophilic groove interacting with the 3′ terminus of ToxI and the 5′ terminus of another ToxI.

Ten nucleotides of ToxI interact with ToxN with large favorable energies for |∆*G_nucleotide-ToxN_*| ≥ 3 kcal/mol (Figure 3B and Table S1). The large favorable gas-phase electrostatic contributions were compensated by the unfavorable polar solvation energy. There were only two nucleotides with favorable electrostatic contribution. The non-polar contribution would be the main driving force for the binding of ToxI to ToxN.

### 2.3. The Function of G5

**Figure 4:**
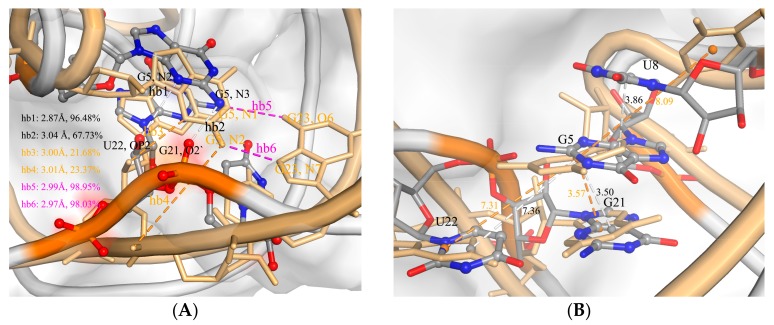
(**A**) The H-bonds formed for G5 in com-ToxI and in apo-ToxI. (**B**) The distances between the mass center of nitrogenous base of U8 and G5. The nucleotides are shown in ball and stick representation and colored with elements in com-ToxI, as well as stick representation in apo-ToxI. The H-bonds were determined by an acceptor···donor distance less than 3.5 Å and an acceptor···H donor angle larger than 120°. Occupancy was defined as the percentage of the snapshots from MD simulation trajectory where a specific hydrogen bond exists.

The crystallographic structure shows that G5 interacts with G21 and U22 by stable hydrogen bonds (H-bonds) between their nitrogenous bases. To evaluate these interactions’ function in the ToxIN complex, we analyzed these H-bonds and calculated the distances between two nitrogenous bases based on our three 1 µs MD simulations for ToxIN, as well as for ToxI. Two nitrogen atoms of the nitrogenous base of G5 formed H-bonds with the oxygen atoms of the backbone of G21, as well as U22. The two H-bonds of G5 (hb1 and hb2) were weaker in com-ToxI than in apo-ToxI (hb3 and hb4) for the rising occupancies, and two new H-bonds (hb5 and hb6) were formed with higher occupancy between G5 and G23 (Figure 4A). The distances between the mass center of the nitrogenous base of G5 and G21/U22 in apo-ToxI were slightly different from those in com-ToxI (Figure 4B). These interactions are key for the stability of the pseudoknot. G5 also interacted with U8 by π-π stacking. The average distance between the mass centers of the nitrogenous base of G5 and U8 in com-ToxI was 3.86 Å, which is much smaller than in apo-ToxI (Figure 4B). These interactions bring out bulged loops, exposing A6 which inserts the hydrophobic pocket formed by residues Phe3, Leu99, Leu100, Leu102, and Leu118 (Figure 3B and Figure S2). The free energy analysis also gave a large binding free energy of −7.66 kcal/mol with favorable nonpolar interaction. This may imply that the loop conformation facilitates its interaction with ToxN rather than located at the energy minimum.

**Figure 5:**
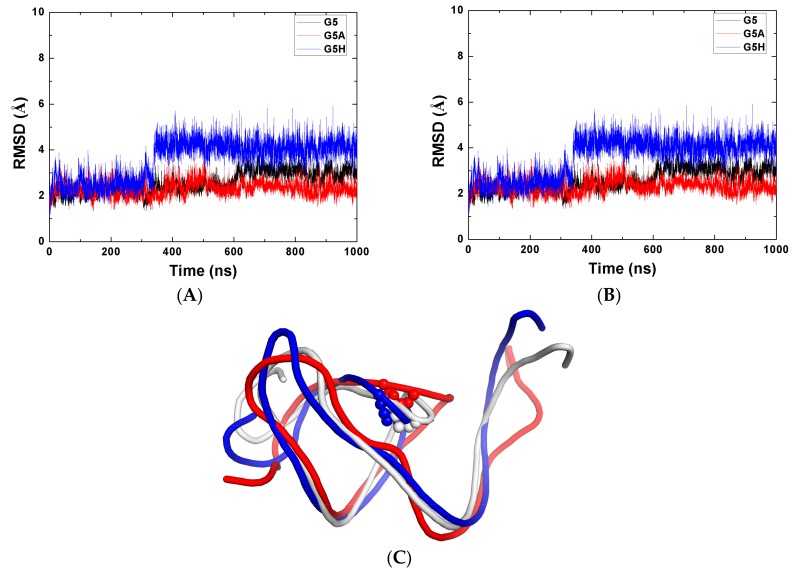
The RMSDs were calculated for the backbone atoms with the nucleotide numbers from 5 to 31 (**A**) relative to their original structure, and (**B**) for the atoms with nucleotides within 5 Å. (**C**) Superimposition of the representative structures of G5 (ToxI monomer in white color), G5A (mutated G5 to A5 in red color), and G5H (a hydrogen atom instead of nucleobase of G5 in blue color). Six atoms, which can show the position of nucleotide 5, are shown in ball representation.

The mutagenesis studies suggested that ToxI loses the antitoxicity in the G5A mutation [11]. To evaluate the structural influences on apo-ToxI, 1 µs MD simulations were carried out for G5A, as well as for G5H with a hydrogen atom instead of the nitrogenous bases of G5. The RMSDs were calculated for the backbone atoms with nucleotide numbers from 5 to 31 relative to their starting structure of MD simulations (Figure 5A), as well as for the atoms which nucleotides within five angstroms (Figure 5B). It is clear that the plot of G5H was different from both of the other systems. The G5A system was in a stable state for the last 300 ns of the MD simulation. The distance between the mass center of the nitrogenous base of A5 and G21 was 3.80Å in G5A, which is a little larger than in G5 (3.60 Å). The same condition was also found for the distance from the mass centers of nitrogenous base of G5/A5 to U22 (6.95 Å in G5 and 7.47 Å in G5A). This all indicates that G5 is much more compact than G5A. The results are also suggested by the average Rg of nucleotides 5–31, which are 12.28 and 12.99 Å in G5 and G5A, respectively. In the G5H, the interaction of nitrogenous base was lost and the average Rg of nucleotides 5–31 was 13.42 Å.

Clustering of trajectories was performed with cpptraj [42] using the hierarchical agglomerative approach clustered on P, O3′, O5′, C3′, C4′, and C5′ atoms of nucleotides using the average distance between members of two clusters. A total of 4000 frames from the last 200 ns were used to cluster with a “sieve” value of 10. The most populated clusters accounted for 52.7%, 74.1%, and 83.6% for G5, G5A, and G5H, respectively. We superimposed the representative frame together as shown in Figure 5C. Large differences of nucleotides conformation were found nearby nucleotide 5, as well as for the few nucleotides in the 3′ and 5′ termini, implying that the mutations only influenced the nearby nucleotides’ conformation when ToxI was in the folded state.

### 2.4. Induced-Fit Mechanism

**Figure 6:**
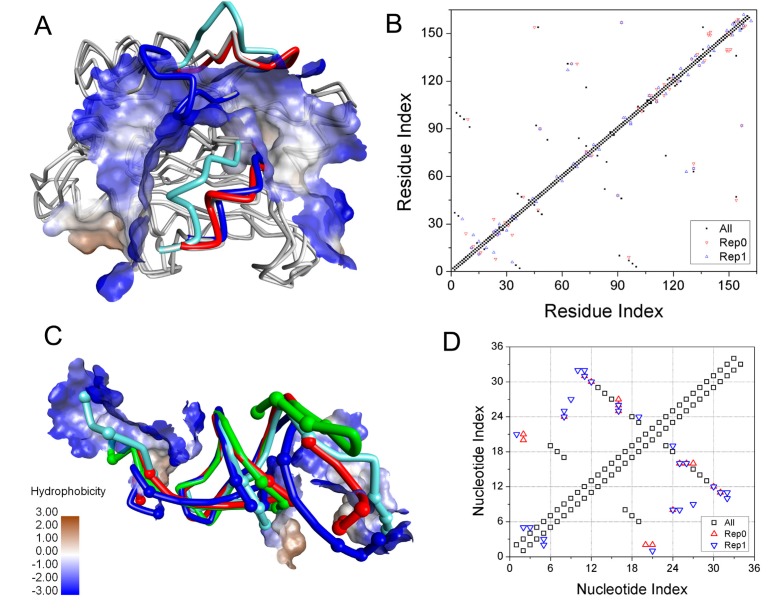
Superimposition of crystallographic structure and the representative structures from the clustering analysis for (**A**) apo-ToxN and (**C**) apo-ToxI. The interaction surfaces between ToxN and ToxI are shown in hydrophobic surface. All the structures are shown in tube representation. The different colors represent different conformations. The crystallographic structures are shown in pale cyan color. The nucleotides with different conformation in apo-ToxI are shown with an atom in a ball representation representing a nucleotide. Contact map based on the representative structures of clusters representative 0 (Rep0) and Rep1 for (**B**) ToxN and (**D**) ToxI Black squares represent the contacts that exist in both Rep0 and Rep1.

Clustering was performed on the combined trajectories—a total of 30,000 frames from three last-500-ns MD simulation trajectories by using a “sieve” value of 10 for apo-ToxI and apo-ToxN. Supplemental Table S2 shows the clustering results. The three most populated clusters accounted for 88.9% and 81.3% of all structures for ToxN and ToxI, respectively. Figure 6 shows the superimposition of the representative structures of the most populated clusters with their crystallographic structure. As shown in Figure 6A,B, the large differences between crystallographic structure and MD structures for ToxN were mainly in residues 23–33 and 53–60, located at the binding pockets.

Up to now, two main hypotheses—“induced-fit” [40,43] and “conformational selection” [44] mechanisms—have been given to explain the coupling of ligand and protein folding. It is clear from Figure 6C,D that the 3′ and 5′ termini of ToxI in the crystallographic structure differed in the three representative structures, is in accordance with the RMSDs analysis. The most favorable nucleotides were from the 3′ and 5′ termini based on the interaction energy analysis. Therefore, the folded apo-ToxI cannot directly bind to ToxN. We can suppose that the ToxN recognizes the ToxI with the “induced-fit” mechanism rather than the “conformational selection” mechanism [23,40,45]. The shift of 3′ and 5′ termini would be the predominant mechanism for ToxI binding to the groove of ToxN with an “induced-fit’ mechanism.

### 2.5. The Dissociation of ToxN(A) from the ToxIN Complex

**Figure 7:**
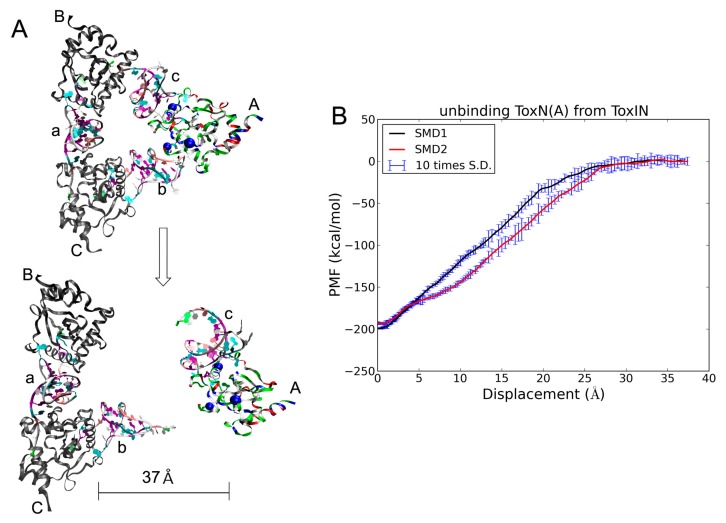
(**A**) The ToxIN in bound state (top) and in the unbound state (bottom). ToxN are shown in ribbons presentation. The dragged ToxN is colored by residue types. The fixed two ToxNs are colored in gray. Three ToxIs are colored by nucleotide name. The three centers are marked as blue balls. (**B**) The potential of mean force (PMF) curves of unbinding ToxN from its complex. SMD: steered molecular dynamics.

The ToxIN complex releases ToxN to realize its bio-function. To evaluate the detailed binding information, we carried out an SMD simulation for ToxN unbinding from the ToxIN complex. In the SMD simulations, ToxN(A) was dragged with three centers and the other two ToxNs were fixed with the Cα atoms (Figure 7A). SMD simulation has been widely used for extensive study of protein mechanical stability and protein-protein binding [38,46]. Previous SMD predictions agreed well with atomic force microscopy (AFM) observations [47]. In the implementation of SMD, the starting structures were rotated for ToxN(A) pointing to the +z direction, and the coordinates of three chosen Cα (Asn59Cα, Tyr115Cα and Asp133Cα) atoms represented the position and orientation of ToxN(A). To evaluate the convergence behavior, two simulation runs were carried out and the PMFs along the displacement are illustrated in Figure 7B. It is clear that the SMDs were convergent. The PMFs increased smoothly, so there would be no clear conformational change during the dissociation.

As shown in Figure 7A, ToxI(c) moved together with the dragged ToxN(A) during the SMD process, and ToxI(b) interacted with the fixed ToxN(C), indicating that ToxN interacted more strongly with the 3′ terminus than with the 5′ terminus. This is in accordance with the binding free energy of each nucleotide. As for ToxI(c), only three of the ten key nucleotides interacted with ToxN(B) versus seven with ToxN(A) (Figure 3B). This also suggests that the stability of the pseudoknot is stronger than the binding between ToxN and the 3′ terminus of ToxI.

**Figure 8:**
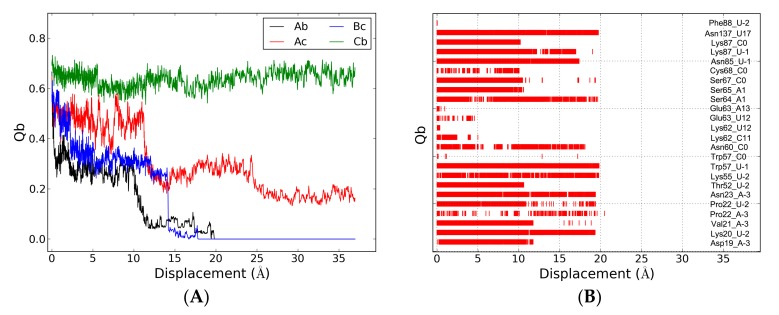
(**A**) Binding contact (Qb) between ToxN and ToxI as a function of displacements. The uppercase and lowercase letters are for ToxN and ToxI, respectively. (**B**) The Qb of residue-nucleotide pair between ToxN(A) and ToxI(b) along the SMD1 simulation.

To investigate the detailed information of dissociation, we calculated the native binding contacts (Qb) using the native contacts patch of the RMSD Trajectory Tool in VMD [48]. Two residues or nucleotides in different monomers are in native binding contact when their heavy atoms are closer than 3.5 Å. Qb(Ac) and Qb(Cb) are the binding contacts between ToxN and the 3′ terminus of ToxI. The plots of Qb(Ac) decreased significantly, but not Qb(Cb) in two SMD simulations (Figure 8A and Figure S3). This can be attributed to the slight fluctuation of ToxN(C) for fixed Cα in the SMD simulation. As shown in Figure 8B, the nucleotides of ToxI(b) involved in the native binding contacts can be roughly divided into two groups. One group (C11, U12, and A13) formed unstable Qb, disappearing at the beginning of 5 Å displacement. The binding free energy analysis also showed that they are not the key nucleotides. The other group with stable Qb was composed of five nucleotides at the 3′ terminus. The Qb of this group reduced obviously at the 10 Å displacement, disappearing completely at 20 Å.

### 2.6. The Dissociation of ToxI(c) from ToxN(A)

**Figure 9:**
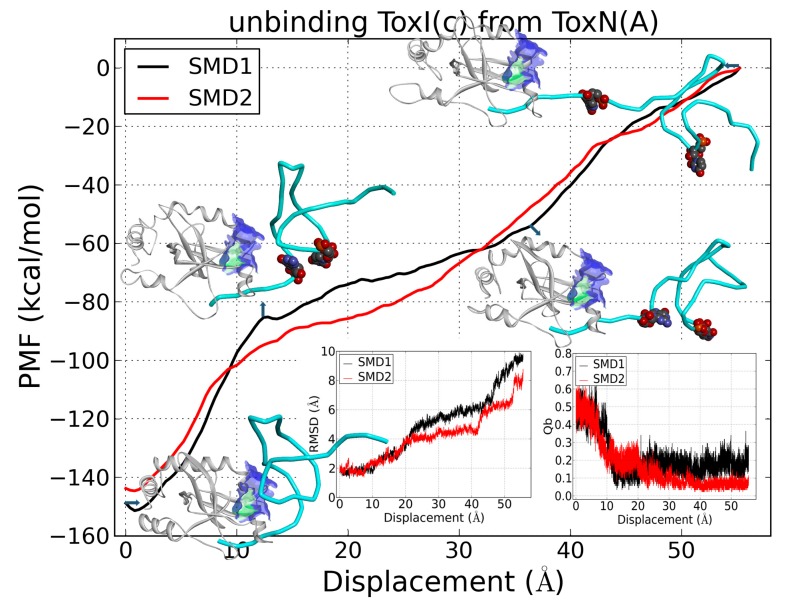
PMF curves of the unbinding of ToxI(c) from ToxN(A) in two SMD simulations. Insets: the Qb between ToxN and ToxI and RMSD of ToxI(c) as a function of displacements, the key structures of SMD1 from trajectories are shown. The structures of ToxN(A) are shown in white color with new cartoon representation, as well as ToxI(c) in cyan with tubes representation. A section of the interacting surfaces is shown. Nucleotides U9 and G26 are shown in ball representation.

To evaluate the dissociation of ToxI(c) from ToxN(A), SMD simulations were also carried out for ToxI(c) dragged away ToxN(A) with two Cα atoms of Glu16 and Leu140 fixed. Three atoms (i.e., C1′ atoms of G6, U13, and U20) were dragged for 56 Å displacement. Figure 9 depicts the PMFs along the displacement for two runs. The unbinding could be roughly divided into three stages according to the PMFs. The first stage was the sharp increase from 2 to 13 Å displacement for PMFs, indicating that a stable structural domain collapsed. The values of Qb gradually decreased from 0.5 to 0.15, and the lines of RMSDs of ToxI were nearly level (Figure 9). Therefore, the collapsed structural domain was a section of the binding between ToxN and ToxI rather than unfolding of ToxI. In this stage, all the binding native contacts disappeared except for those involved in three nucleotides (A33, A32, G31) of the 3′ terminus. The second stage was from 13 to 37 Å displacement, which showed a slight increase for PMFs. In this stage, ToxI was far away ToxN when the 3′ terminus was pulled tightly. The last stage was from 37 Å to the end of SMD simulation. In this stage, the pseudoknot of ToxI started to unfold, for the RMSDs increased clearly and the distance between nucleotides U9 and G26 increased by 17 Å from 23 to 40 Å in SMD1 (Figure S4).

**Figure 10:**
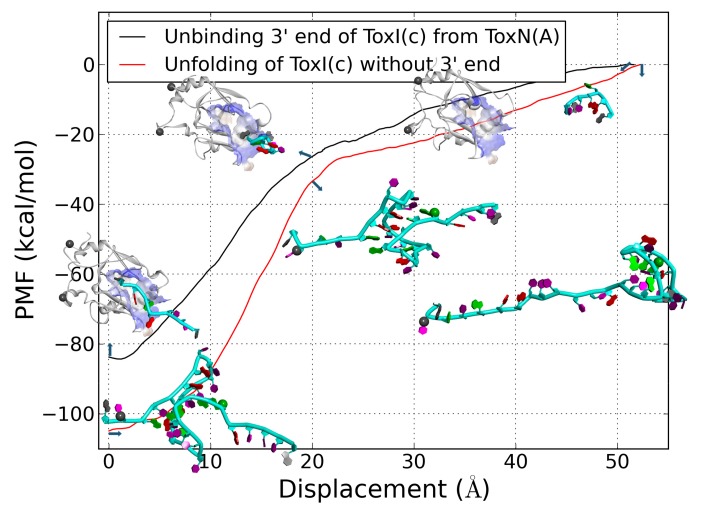
PMF curves of the unbinding of the 3′ terminus of ToxI(c) from ToxN(A) and the unfolding of ToxI(c) without the 3′ terminus. Insets: the starting, at displacement 20 Å, and the last structures are shown. The structures of ToxN(A) are shown in white color with new cartoon representation, as well as ToxI(c) in cyan with tubes representation and the nucleobase with rings. The atoms fixed in the SMD simulations are shown in grey color with balls.

As shown in Figure 9, the ToxI(c) 3′ terminus was still bound to ToxN(A) after an SMD simulation of 56 Å displacement. However, the SMD should be terminated because the water box was not large enough to hold the entire system for more pulling displacements. If we added the water box size, the more atoms in system would increase the computational cost. So, we divided the ToxN(A)-ToxI(c) system into two subsystems for small size according to the last structure from SMD1 of ToxN(A)-ToxI(c). One was the ToxN(A) and seven nucleotides from the 3′ terminus. The other was the ToxI(c) without the 3′ terminus. Figure 10 shows the PMF curves of unbinding and unfolding. The 3′ terminus of ToxI(c) moved out of the binding package of ToxN(A) at the 20 Å displacement with a sharp increase of PMF. After that, the PMF increased slowly because there was some temporary interaction. As for the unfolding, the PMF increased obviously from 5 to 23 Å displacement. In this stage, the helix near the 3′ terminus unfolded first, and the stable triplex structure was destroyed (Figure 10). After 23 Å displacement, the PMF increased slowly with a large conformational change. The entire unfolding roughly followed an unzipping mechanism. The unfolding process described here is in accordance with Wang’s work about pseudoknotted RNA unfolding [49]. So, the triplex structure is key for the unfolding of ToxI. The differences between the minimum and maximum of PMF were 73 and 57 kcal/mol for unfolding of the triplex structure in ToxI and the unbinding of seven nucleotides from the binding package of ToxN, respectively. Although the differences were larger than the real values, the condition for the unbinding SMD simulations were the same as the unfolding. These results may indicate an easy unbinding relative to the unfolding of ToxI [50].

## 3. Modelling and Simulation

### 3.1. Molecular Dynamics Simulations

The initial structures of the MD simulations were extracted from the crystal structure of the ToxIN complex determined by Blower et al. (PDB ID: 2xdd) [11]. Independent MD simulations were carried out for the whole complex (ToxIN), ToxN and ToxI monomers (apo-ToxN and apo-ToxI), and the mutations of nucleotide G5 to A5 in ToxI monomer (G5A) as well as a hydrogen atom instead of the nitrogenous bases of G5 (G5H). SMD simulations were also performed for the ToxIN, protein ToxN(A), and RNA ToxI(c) complex (ToxN(A)-ToxI(c)), and two subsystems, ToxN(A)-3′ terminus of ToxI(c) complex and ToxI(c) monomer. Table 1 lists the detailed information of these simulations, including total number of atoms, duration of the MD simulations, and the number of MD simulations.

The crystallographic water molecules within 5 Å of system were retained. The hydrogen atoms were added according to each residue’s or nucleotide’s default protonation states at a neutral pH. All the systems were solvated in rectangular TIP3P water [51] boxes with the minimum solute–box boundary distance set to 10 Å. The appropriate numbers of counter-ions were added to neutralize the systems. The AMBER force field (FF12SB) [52,53] was used to describe the protein and RNA parameters, ions, and water molecules. The force field parameter of mutated nucleotide G5H was generated according to G5 parameters and modified the atoms’ charge based on the RESP method [54] with the Antechamber module of the AMBER12 package.

The AMBER12 package [55] was used to perform all energy minimizations and MD simulations. We performed a two-step extensive energy minimization process by the steepest descent method and the conjugate gradient algorithm to relieve the bad contacts and to direct each system toward energetically favorable conformations. After minimization, each system was heated from 0 to 300 K over 70 ps, applying harmonic restraints with force constants of 2 kcal/(mol·Å^2^) to all solute atoms. To adjust the solvent density, a constant-pressure MD simulation was carried out for 90 ps. Finally, constant-pressure MD simulation without any restraints for each system was carried out at 300 K with a Langevin thermostat and a target pressure 1.0 atm using isotropic positional scaling. The particle mesh Ewald method [56] was applied to treat the long-range electrostatic interactions with a cutoff of 12 Å for all MD simulations. The SHAKE algorithm [57] was employed to treat the covalent bonds involving hydrogen atoms, and the time step was set to be 2 fs.

### 3.2. Residue-Inhibitor Interaction Decomposition

To understand the ToxN and ToxI interaction in more detail, the interaction energies between ToxN and each nucleotide of ToxI (as well as between ToxI and each residue of ToxN) were calculated using the theory of free energy decomposition [58]. This approach has been successful in the analysis of the interaction between protein and ligand or between protein and protein [59,60,61]. The binding interaction of each pair (∆*G_ToxN-nuc/ToxI-res_*) includes four terms: van der Waals energy (∆*E_vdw_*), electrostatic energy (∆*E_ele_*), polar solvation contribution (∆*G_pol_*), and nonpolar solvation contribution (∆*G_nonpol_*):

∆*G_ToxN-nuc/ToxI-res_* = ∆*E_ele_* + ∆*E_vdw_* + ∆*G_pol_* + ∆*G_nonpol_* (1)

All energy components were calculated using 150 snapshots extracted evenly from the last 300 ns of the MD simulation. ∆*E_vdw_* and ∆*E_ele_* were calculated with the same parameter as the MD simulation, and ∆*G_pol_* was calculated by generalized Born (GB) methods implemented in SANDER. ∆*G_nonpol_* was determined with the equation ∆*G_nonpol_* = γ*SASA* + β, where SASA is the solvent-accessible surface area that was determined using the LCPO model [62]. The values γ and β are the empirical constants, and were set 0.005 kcal/(mol·Å^2^) and 0, respectively [63].

### 3.3. SMD and PMF in 3nD

The SMD simulations were performed with the modified NAMD 2.13 [64,65] with a larger margin of water box than the equilibrated MD simulation in the steered direction. The force field was the same as the equilibrated MD simulation. The systems were put in a box of water and neutralized. The displacement for the whole SMD simulation was divided into multi-windows, every 0.1 Å from 0 to 5 Å, every 0.2 Å from 5 to 13 Å, and every 0.4 Å from 13 Å to the end. At each of the displaced positions, 2 ns equilibrated MD simulations were carried out with the steered centers and selected atoms fixed, then four sets of data of the forces acting on the steered centers were collected from four segments of unbiased MD runs in order to obtain the PMFs with the TI3nD method [64,66,67].

The PMF in 3n dimensions (3nD), *W*[*r*_1_, *r*_2_, …, *r_n_*], is a function of the 3n coordinates of *n* centers (*r*_1_, *r*_2_, …, *r_n_*) along a single curve/line *u*(*λ*) = (*r*_1_(*λ*), *r*_2_(*λ*), …, *r_n_*(*λ*)) connecting one state *u*(0) = (*r*_10_, *r*_20_, …, *r*_*n*0_) that is arbitrarily chosen from the bound state ensemble to the corresponding one state *u*(1) = (*r*_1∞_, *r*_2∞_, …, *r*_*n*∞_) that belongs to the dissociated state ensemble. The 3nD PMF difference between two states is equal to the line integral of the mean force acting the 3n degrees of freedom along a line connecting the two states. Since PMF is a function of state, any one line/curve connecting the two end states is necessary and sufficient for the computation in a straightforward extension of the well-known thermal integration thermodynamic integration in 3nD (TI3nD) given by the following formula [64,66,67]:

(2) $$W\left[r_{10},r_{20},\ldots r_{n0}\right]-W\left[r_{1\infty },r_{2\infty },\ldots r_{n\infty }\right]=\int_{\lambda =0}^{\lambda =1}du\left(\lambda \right)\cdot {\langle -\frac{\partial H}{\partial u}\rangle }_{u(\lambda )}$$

where *H* is the Hamiltonian of the entire system, a function of 3*N* coordinates (*r*_1_, *r*_2_, …, *r_n_*; *r*_*n*+1_, *r*_*n*+2_, …, *r_N_*) of all the *N* atoms of a model system.

## 4. Conclusions

Long time MD simulations were used to study the stability of ToxN and ToxI monomers, as well as their complex. The results suggest that the monomers are less stable upon their complexation, especially for the 3′ and 5′ termini of ToxI. The stable H-bonds of nucleotide G5 formed with G21 and U22 in com-ToxI are adjusted in apo-ToxI. To evaluate the function of G5 on stability of ToxI, the MD simulations of mutation G5A and G5H indicate a conformational difference of nearby nucleotides effected by the mutation G5A. The flexible 3′ and 5′ termini adjust their conformation to match the groove of ToxN during the process of binding with an “induced-fit” binding mechanism. The energetic calculation shows nineteen key residues of ToxN and ten nucleotides of ToxI in the interaction between ToxN and ToxI. The SMD simulations combined with TI3nD method were applied to obtain the PMF and provided atomistic pictures for the unbinding of ToxIN complex. The unbinding follows five steps. Firstly, the 5′ terminus of ToxI dissociates from ToxN. Secondly, the interactions involved in the 3′ terminus of ToxI without three nucleotides (A33, A32, G31) are lost. Thirdly, ToxI start to unfold. Then three nucleotides (A33, A32, G31) of ToxI leave the binding pocket of ToxN. Lastly, ToxN is unfolded.

## Figures and Tables

**Table 1:** Systems for molecular dynamics (MD) simulations.

System	Methods	Total Number of Atoms	MD Length (ns)
ToxIN	Equilibrated MD	93,408	1000 × 3 = 3000
ToxI	Equilibrated MD	26,478	1000 × 3 = 3000
ToxN	Equilibrated MD	19,351	1000 × 3 = 3000
G5A	Equilibrated MD	17,016	1000
G5H	Equilibrated MD	16,349	1000
ToxIN	SMD	115,152	338.8 × 2 = 677.6
ToxN(A)-ToxI(c)	SMD	50,534	338.8 × 2 = 677.6
ToxN(A)-3′ terminus of ToxI(c)	SMD	43,306	338.8
ToxI(c)	SMD	38,519	338.8

## Supplementary Materials

Supplementary materials can be found at http://www.mdpi.com/1422-0067/19/11/3524/s1.
