# Supplementary material for: Atomistic Analysis of ToxN and ToxI Complex Unbinding Mechanism
Source: Int J Mol Sci. 2018 Nov 9;19(11):3524. doi: 10.3390/ijms19113524 (PMC6275071; doi:10.3390/ijms19113524)
Supplement: Supplementary file 1 [file ijms-19-03524-s001.pdf]

Table S1. Energy terms of per residue/nucleotide calculated by free energy decomposition method (kcal/mol).

Items	Residues/Nucleotide	$\Delta E_{vdw} + \Delta G_{nonpol}$	$\Delta E_{ele} + \Delta G_{pol}$	$\Delta G_{ToxN\text{-}nuc/ToxI\text{-}res}$
ToxI	Lys2	-3.03	-4.9	-7.93
	Lys20	-1.58	-4.62	-6.2
	Lys33	-4.3	-2.05	-6.35
	Lys55	-0.61	-4.99	-5.6
	Lys62	-2.39	-7.36	-9.75
	Glu63	-2.75	7.91	5.16
	Lys70	-0.18	-7.25	-7.43
	Glu73	-1.69	8.64	6.95
	Glu78	-1.21	7.51	6.3
	Asn79	-3.47	-1.84	-5.31
	Lys87	-0.91	-13.73	-14.64
	Arg112	-3.57	-5.86	-9.43
	Met113	-5.71	-0.15	-5.86
	Lys116	-2.29	-5.15	-7.44
	Arg122	-5.04	-22.7	-27.74
	Asp126	-0.14	7.17	7.03
	Lys127	-0.49	-7.18	-7.67
	Lys131	-0.34	-4.97	-5.31
	Asp133	-0.15	5.75	5.6
ToxN	A-3	-9.31	5.59	-3.72
	U-1	-5.94	1.77	-4.17
	A1	-9.39	6.04	-3.35
	A6	-13.46	5.8	-7.66
	U7	-7.33	2.34	-4.99
	U8	-1.84	-2.19	-4.03
	C27	-1.74	-4.81	-6.55
	G30	-10.48	0.72	-9.76
	A31	-11.73	7.3	-4.43
	A32	-16.21	8.63	-7.58

Table S2. Clustering Results for all Clusters.

Items	Cluster	Frames	%	AvgDist[a]	Stdev[a]	AvgCDist[b]
ToxN	0	14095	47.0	1.86	0.33	2.56
	1	10001	33.3	1.69	0.34	2.63
	2	2565	8.6	1.60	0.28	2.54
	3	1889	6.3	1.78	0.36	2.47
	4	1302	4.3	1.57	0.38	2.52
	5	148	0.5	1.14	0.13	3.14
ToxI	0	10075	33.6	3.83	0.94	5.79
	1	8051	26.8	2.82	0.81	6.80
	2	6266	20.9	3.07	0.93	6.37
	3	2753	9.2	3.08	0.72	5.83
	4	1070	3.6	3.25	0.89	5.84
	5	935	3.1	2.56	0.77	5.97
	6	694	2.3	3.03	0.78	5.37
	7	156	0.5	2.94	0.78	6.45

[a]AvgDist and Stdev are the average distance and standard deviation (in Å) between all frames in the cluster, respectively. [b]AvgCdist is the average distance (in Å) of the cluster to every other cluster.

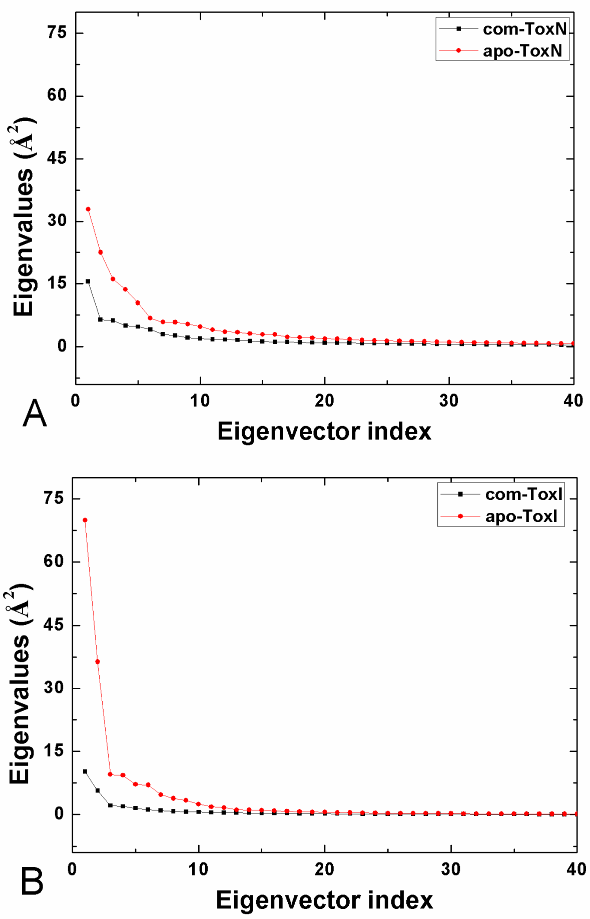

Figure S1. Comparison of the eigenvalues plotted against the corresponding eigenvector indices obtained from the covariance matrix constructed from the equilibrium phase of MD simulations for ToxN (A), as well as for ToxI (B).

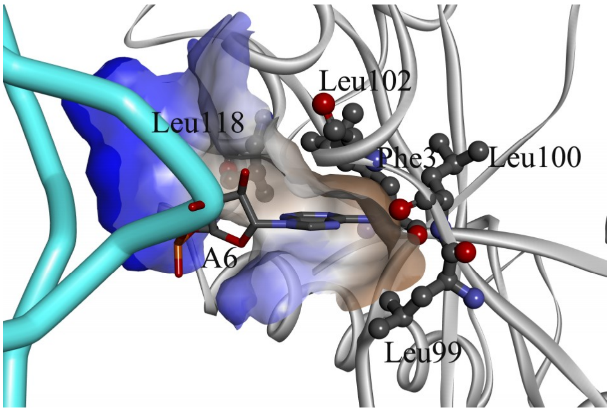

Figure S2. The hydrophobic pocket formed by residues Phe3, Leu99, Leu100, Leu102 and Leu118.

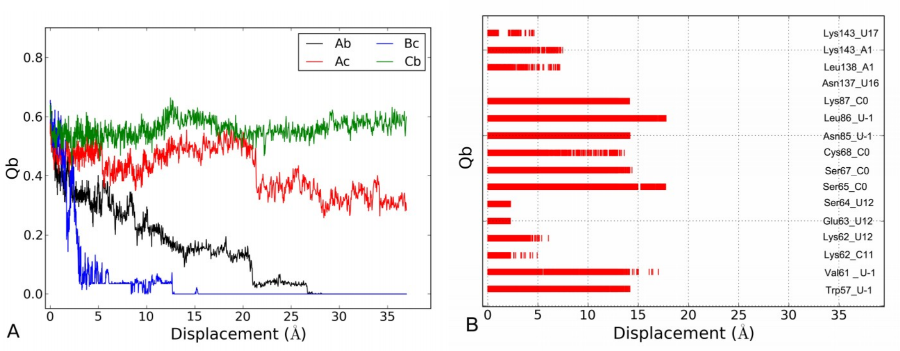

Figure S3. (A) Qb between ToxN and ToxI as a function of displacements in SMD2. (B) The Qb of residue-nucleotide between ToxN(B) and ToxI(c)pair along the SMD1 simulation.

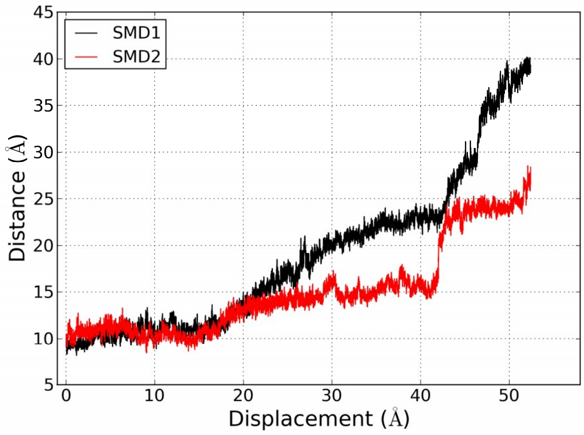

Figure S4. The distances between nucleotide U9 and G26 as a function of displacements in two SMDs.
